# Cholecystectomy can increase the risk of colorectal cancer: A meta-analysis of 10 cohort studies

**DOI:** 10.1371/journal.pone.0181852

**Published:** 2017-08-03

**Authors:** Yong Zhang, Hao Liu, Li Li, Min Ai, Zheng Gong, Yong He, Yunlong Dong, Shuanglan Xu, Jun Wang, Bo Jin, Jianping Liu, Zhaowei Teng

**Affiliations:** 1 Department of General Surgery, The 6th Affiliated Hospital of Kunming Medical University, The People’s Hospital of Yuxi City, Yuxi, Yunnan, China; 2 School of Public Health, Dali University, Dali, Yunnan, China; 3 Department of Respiratory Medicine, The Affiliated Yanan Hospital of Kunming Medical University, Kunming, Yunnan, China; 4 Department of Science and Education, The 6th Affiliated Hospital of Kunming Medical University, The People’s Hospital of Yuxi City, Yuxi, Yunnan, China; 5 Department of Orthopedic Surgery, The 6th Affiliated Hospital of Kunming Medical University, The People’s Hospital of Yuxi City, Yuxi, Yunnan, China; University of Arizona, UNITED STATES

## Abstract

**Objective:**

This study aimed to elucidate the effects of cholecystectomy on the risk of colorectal cancer (CRC) by conducting a meta-analysis of 10 cohort studies.

**Methods:**

The eligible cohort studies were selected by searching the PubMed and EMBASE databases from their origination to June 30, 2016, as well as by consulting the reference lists of the selected articles. Two authors individually collected the data from the 10 papers. When the data showed marked heterogeneity, we used a random-effects model to estimate the overall pooled risk; otherwise, a fixed effects model was employed.

**Results:**

The final analysis included ten cohort studies. According to the Newcastle-Ottawa Scale (NOS), nine papers were considered high quality. After the data of these 9 studies were combined, an increased risk of CRC was found among the individuals who had undergone cholecystectomy (risk ratio (RR) 1.22; 95% confidence interval (CI) 1.08–1.38). In addition, we also found a promising increased risk for colon cancer (CC) (RR 1.30, 95% CI 1.07–1.58), but no relationship between cholecystectomy and rectum cancer (RC) (RR 1.09; 95% CI 0.89–1.34) was observed. Additionally, in the sub-group analysis of the tumor location in the colon, a positive risk for ascending colon cancer (ACC) was found (RR 1.18, 95% CI 1.11–1.26). After combining the ACC, transverse colon cancer (TCC), sigmoid colon cancer (SCC) and descending colon cancer (DCC) patients, we found a positive relationship with cholecystectomy (RR 1.18, 95% CI 1.11–1.26). Furthermore, after combining the ACC and DCC patients, we also found a positive relationship with cholecystectomy (RR 1.28; 95% CI 1.11–1.26) in the sub-group analysis. In an additional sub-group analysis of patients from Western countries, there was a positive relationship between cholecystectomy and the risk of CRC (RR 1.20; 95% CI 1.05–1.36). Furthermore, a positive relationship between female gender and CRC was also found (RR 1.17; 95% CI 1.03–1.34). However, there was no relationship between gender and CC or RC. Furthermore, no publication bias was observed, and the sensitivity analysis indicated stable results.

**Conclusions:**

This meta-analysis of 10 cohort studies revealed that cholecystectomy is associated with an increased risk for CRC, CC and ACC, particularly in Western countries. No relationship between cholecystectomy and RC was observed. There was no relationship between gender and either CC or RC, but a positive relationship between female gender and CRC was observed.

## Introduction

According to the American Cancer Society, colorectal cancer (CRC) is the third leading cause of morbidity and mortality in the United States (US) [1]. In Europe, CRC is the fourth leading cause of cancer-related deaths [2], and the specific incidence rate (SIR) and specific mortality rate (SMR) of CRC is the highest in the World Health Organization (WHO) European region [3]. Several studies have demonstrated that CRC is still a major health problem [4–7], and the cancer burden of CRC is expected to increase over the next several decades [2] in both developed and developing countries. However, the etiology and causes of CRC are numerous and unclear [8–10].

Due to improvements in living conditions and lifestyle changes, gallbladder diseases (GBDs), such as cholecystitis, cholelithiasis, and gallbladder polyps, are common and costly [11], with an estimated 380,000 GBD patients seeking treatment from physicians each year [12]. Currently, cholecystectomy, particularly laparoscopic cholecystectomy (LC), is the standard therapy for GBD worldwide [13].

Both the biliary tract system and gastrointestinal system are part of the digestive system. Hence, previous studies [14–16] indicated that the incidence of CRC was increased among individuals who underwent cholecystectomy. However, other studies [17, 18] have suggested conflicting results, indicating that there was no relation between cholecystectomy and CRC. One study from Japan [19] revealed a negative correlation between cholecystectomy and large bowel carcinoma. Another meta-analysis [20] discussed the relation between cholecystectomy and large bowel carcinoma by separately analyzing cohort studies and case-control studies, but the results were also conflicting. Based on the above controversial results, we performed a meta-analysis to further explore the relationship between cholecystectomy and the risk of large bowel carcinoma. In this study, we abided by the meta-analysis of observational studies in epidemiology (MOOSE) guidelines [21].

## Materials and methods

### Search strategy and data sources

The cohort studies representing the impact of cholecystectomy on the risk of CRC were searched from the PubMed and EMBASE databases (from their origination to June 30, 2016) without restrictions. To identify any additional studies, the reference lists of the relevant articles were also searched. We used the following Entry Terms and Medical Subject Headings (Mesh): (i) "Neoplasms, Colorectal", "Colorectal Neoplasm", "Neoplasm, Colorectal", "Colorectal Tumors", "Colorectal Tumor", "Tumor, Colorectal", "Tumors, Colorectal", "Colorectal Carcinoma", "Carcinoma, Colorectal", "Carcinomas, Colorectal", "Colorectal Carcinomas", " Colorectal Cancer ", " Cancer, Colorectal ", "Cancers, Colorectal", "Colorectal Cancers", OR "Colorectal Neoplasms" [Mesh]; and (ii) ("Cholecystectomies" OR "Cholecystectomy" [Mesh]) OR ("Laparoscopic Cholecystectomies", "Cholecystectomies, Laparoscopic", "Cholecystectomy, Celioscopic", "Celioscopic Cholecystectomies", "Cholecystectomies, Celioscopic", "Celioscopic Cholecystectomy", OR "Cholecystectomy, Laparoscopic" [Mesh]); and (iii) "Cohort Study" OR "Cohort Studies" [Mesh].

### Study selection

The studies were included in the meta-analysis if they satisfied all of the following criteria: (a) original data source in a cohort study; (b) examined the effects of cholecystectomy on the incidence of CRC, colon cancer (CC) or rectum cancer (RC); (c) the outcome of interest was the incidence of large intestine carcinoma; (d) the exposure of interest was open or laparoscopic cholecystectomy; and (e) the adjusted relative risks (RRs) or standardized incidence ratio (SIR) and the corresponding 95% confidence intervals (CIs) or data to calculate them were provided. All of the reference lists of the retrieved articles were searched by hand to identify any additional studies. Two authors individually examined all of the studies selected from the databases. If the population was studied in more than one study or the data were duplicated, the article with the most comprehensive outcome evaluation or/and the largest sample size was utilized.

### Data extraction and quality assessment

According to the abovementioned selection criteria, the two authors (HL and YZ) separately evaluated all of the retrieved studies. In addition, a cross-reference search of eligible studies was performed to identify any articles that were not found during the database search. The following information was extracted from each eligible study: the first author’s name; publication year; study region; study period; sample size; number of cases; gender; age; follow-up time; SIR or RR and the 95% CI; and confounding factors. Any disagreements were resolved by discussion or consultation with the co-corresponding authors (LL and ZWT). The Newcastle-Ottawa Scale (NOS) was used to assess the methodological quality of the included studies [22]. The NOS contains three parameters of quality: selection, comparability, and outcome (cohort studies). The maximum NOS score is 9. We defined studies with an NOS score <7.0 as low quality and an NOS score ≥7.0 as high quality.

### Statistical analyses

The association between cholecystectomy and the risk of CRC was estimated by computing the pooled RRs and their 95% CI, which were calculated from the adjusted RRs or SIRs and 95% CIs reported in the studies. In this meta-analysis, the SIR was deemed equivalent to the RR [23]. The heterogeneity of the studies was determined using the Q test and the I^2^ test [24]. When the heterogeneity of the included studies was significant, we employed the D-L random-effects model [25] as the pooling method; otherwise, the M-H fixed effects model [26] was adopted. To uncover the source of heterogeneity thoroughly, we also conducted sub-group analyses performed by gender, CC, RC, the tumor location in the colon, and geographic location of the study sample. Additionally, Begg’s funnel plots and Egger’s regression tests [27] were introduced to assess the potential for publication bias. The data analyses were performed using Stata version 13.1 (StataCorp LP, College Station, TX, USA).

## Results

### Literature search and study characteristics

**Fig 1:**
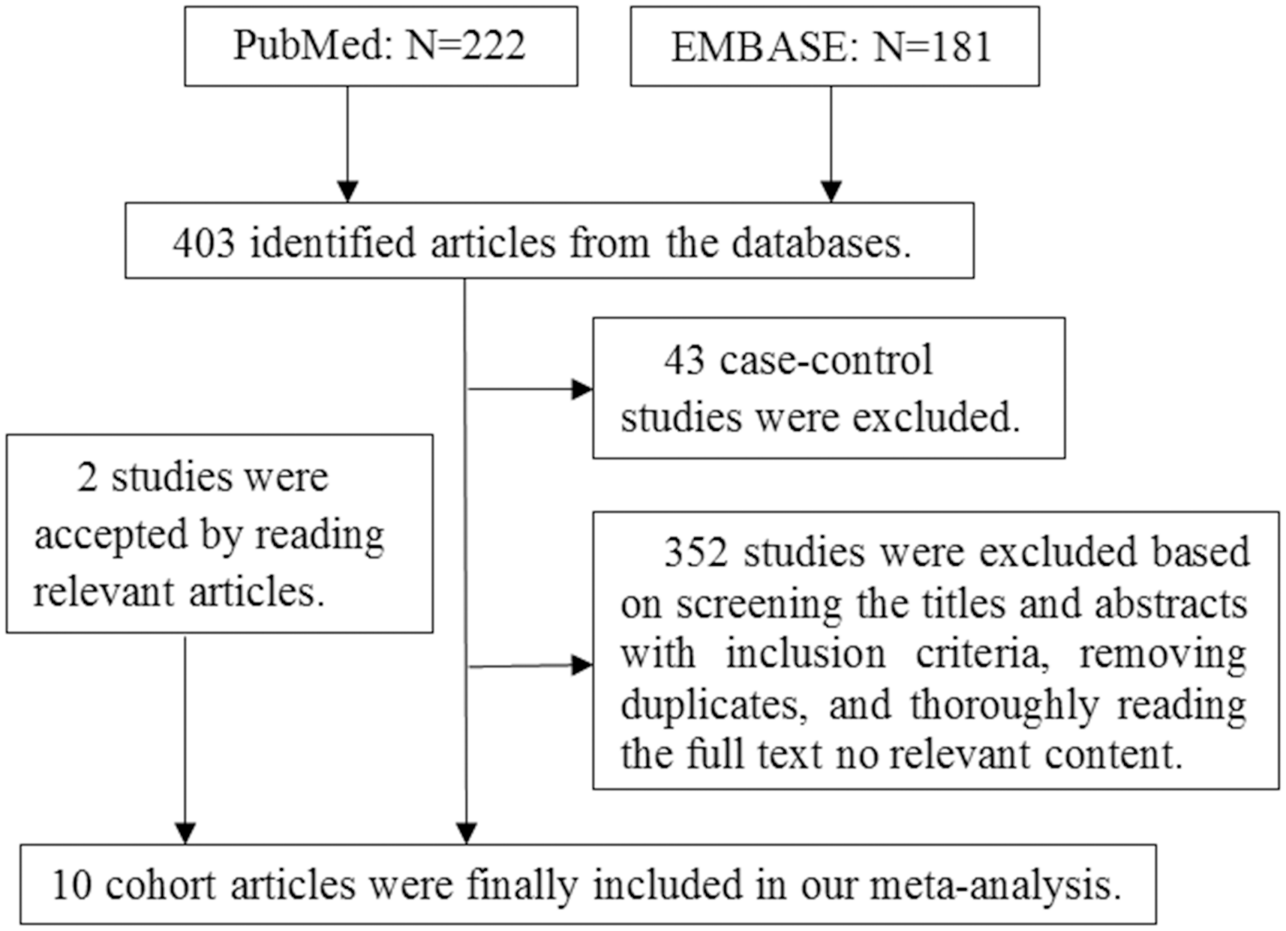
Flow chart illustrating the literature search for cohort studies on cholecystectomy in relation to CRC. Note: CRC represents colorectal cancer.

As shown in Fig 1, a total of 403 articles (222 from PubMed and 181 from EMBASE) were identified in the original search. Among these articles, 43 case-control studies were first excluded. In total, 352 articles were excluded after reviewing the titles and abstracts, removing duplicates, and thoroughly reading the full text. Furthermore, 2 papers [28, 29] were included after reading the relevant articles [30, 31]. Finally, ten cohort studies were included in our final analysis. Six of the 10 cohort studies [32–37] were from Europe, three of the articles were from America [28, 29, 38], and one article was from China [39]. The main characteristics and the quality scores of the ten studies are described in Table 1. The NOS scores for 7 of the included articles were ≥7.0, indicating high quality.

**Table 1:** Characteristics of the 10 cohort studies.

Author, year, country, study period	Sample size	Age: Mean or Range	Cases	Follow-up years	Exposure ascertainment	Outcome ascertainment	Effect estimate (RR, 95% CI)			Adjustments	NOS
							CRC	CC	RC		
Linos, D., 1981, USA, 1950–1969	1,681	NA	42	1-29(13)	Hospital database	Hospital records and self-report	1.3 (0.7–2.2) m	0.9 (0.3–1.9) CC m	2.3 (0.9–4.8) m	Age, Sex, Stage of colon cancer	7
							1.3 (0.9–1.9) w	1.7 (1.1–2.5) CC w	0.5 (0.1–1.3) w		
								1.7 (0.5–4.3) AC m			
								2.1 (1.1–3.6) AC w			
Turunen, M.J., 1981,Helsinki,1969–1977	13,822	NA	304	11	Hospital database	Autopsy examination	1.59 (1.01–2.55)	3.00 (NA) Rc	NA	Age, sex	6
								1.20 (NA) Lc			
								1.31 (NA) Sc			
Adami, H.O., 1983, Sweden, 1964–1978	16,773	51.5±14.2 m 45.6±16.5 w 47.4±16.0 t	130 (46 m;84 w)	11–14	Health and welfare inpatient register	ICD code	0.79 (0.58–1.05) m	0.80 (0.52–1.17) m	0.77 (0.48–1.20) m	Age, sex	8
							0.88 (0.70–1.09) w	1.04 (0.80–1.34) w	0.63 (0.40–0.94) w		
							0.85 (0.68–1.07) t	0.95 (0.76–1.18) t	0.69 (0.50–0.93) t		
Nielsen, G.P., 1991,Iceland,1955–1980	3,425	59.5 m	57	8–33	Icelandic Cancer Registry	Diagnosed	1.38 (0.83–2.15) m	1.74 (0.99–2.82) m	0.65 (0.13–1.19) m	Age, sex, cancer site, and calendar year	8
		53.7 w					0.96 (0.69–1.31) w	0.95 (0.64–1.37) w	0.98 (0.45–1.86) w		
Goldbohm, R.A., 1993, Netherlands,1983–1986	120,852	55–69	478	3.3	Cancer registry; The PALGA registry	ICD-Oncology codes	1.78 (1.03–3.08) m	1.66 (0.61–4.52) Rc m	1.70 (0.73–3.94) m	No cholecystectomy, age, large bowel cancer in first-degree relatives, Quetelet index, parity (w), intake of energy and alcohol and fat (m), meat protein (m) and dietary fiber	7
							1.51 (1.02–2.23) w	1.89 (1.04–3.42) Rc w	1.55 (0.73–3.27) w		
								2.22 (0.90–5.46) Lc m			
								1.25 (0.60–2.59) Lc w			
Schernhammer, E.S, 2003, USA,1982–1998	85,184 w	50.8 (36–61)	133	16	The Nurses’ Health Study	Questionnaire, diagnosed	1.19 (0.98–1.44)	1.35 (0.97–1.88) Proximal CC	1.58 (1.05–2.36)	Age, smoking history, height, weight, physical activity, aspirin use, menopausal status, postmenopausal hormone use, family history of CRC, special screen, diet	7
								0.95 (0.64–1.43) Distal CC			
								1.17 (0.91–1.51) Total CC			
Goldacre, MJ, 2005,England, 1963–1999	39,254	15–84	320/CC	≥10	Oxford record-linkage study	ICD code	NA	1.01 (0.90–1.12)	1.04 (0.89–1.20)	Cancer incidence rates, age, sex, calendar year, residence district, excluding cancers in the first 2 years after admission for cholecystectomy or reference conditions	6
			185/RC								
Shao, T., 2005,England,1987–2002	77,201	≥40	2,515	15	GPRD	Diagnosed	1.43 (1.18–1.73) m	1.76 (1.40–2.21) m	0.98 (0.77–1.25) m	Age, sex, BMI, smoke, HRT, NSAID use, T2DM, calcium use, HMG-CoA reductase inhibitor use, diet	8
							1.23 (1.05–1.45) w	1.35 (1.12–1.63) w	1.01 (0.81–1.25) w		
							1.32 (1.16–1.48) t	1.51 (1.30–1.74) t	1.00 (0.85–1.17) t		
Hartz, A., 2012, USA, 1993–1998	150,912 w	50–79	1,489	8	National database	Self-report	1.36 (1.13–1.64)	NA	NA	Age, smoking, diet, obesity, hormone therapy, use aspirin and NSAIDs, family history, comorbidities	7
Chen, Y.K., 2014, China, 2000–2010	15,545	55	551	11	NHI system	ICD code	1.56 (1.12–2.17)	2.98 (1.08–8.21) AC	2.46 (1.13–5.39)	Index date, age, sex	7
								1.61 (0.10–26.2) TCC			
								NA DCC			
								1.47 (0.39–5.50) SCC			

Notes: m: men; w: women; t: total; NA: data not applicable; CRC: colorectal cancer; CC: colon cancer; ACC: ascending colon cancer; TCC: transverse colon cancer; DCC: descending colon cancer; SCC: sigmoid colon cancer; RC: rectum cancer; Rc: right colon; Lc: left colon; Sc: sigmoid colon; Special screen: previous examination by colonoscopy or sigmoidoscopy and the indications of the procedure; GPRD: the General Practice Research Database; BMI: body mass index (kg/m^2^); HRT: hormone replacement therapy; the PALGA registry may represent the Dutch national database of pathology reports, but no statement was included in the article. NSAIDs: non-steroidal anti-inflammatory drugs.

### Meta-analysis

**Fig 2:**
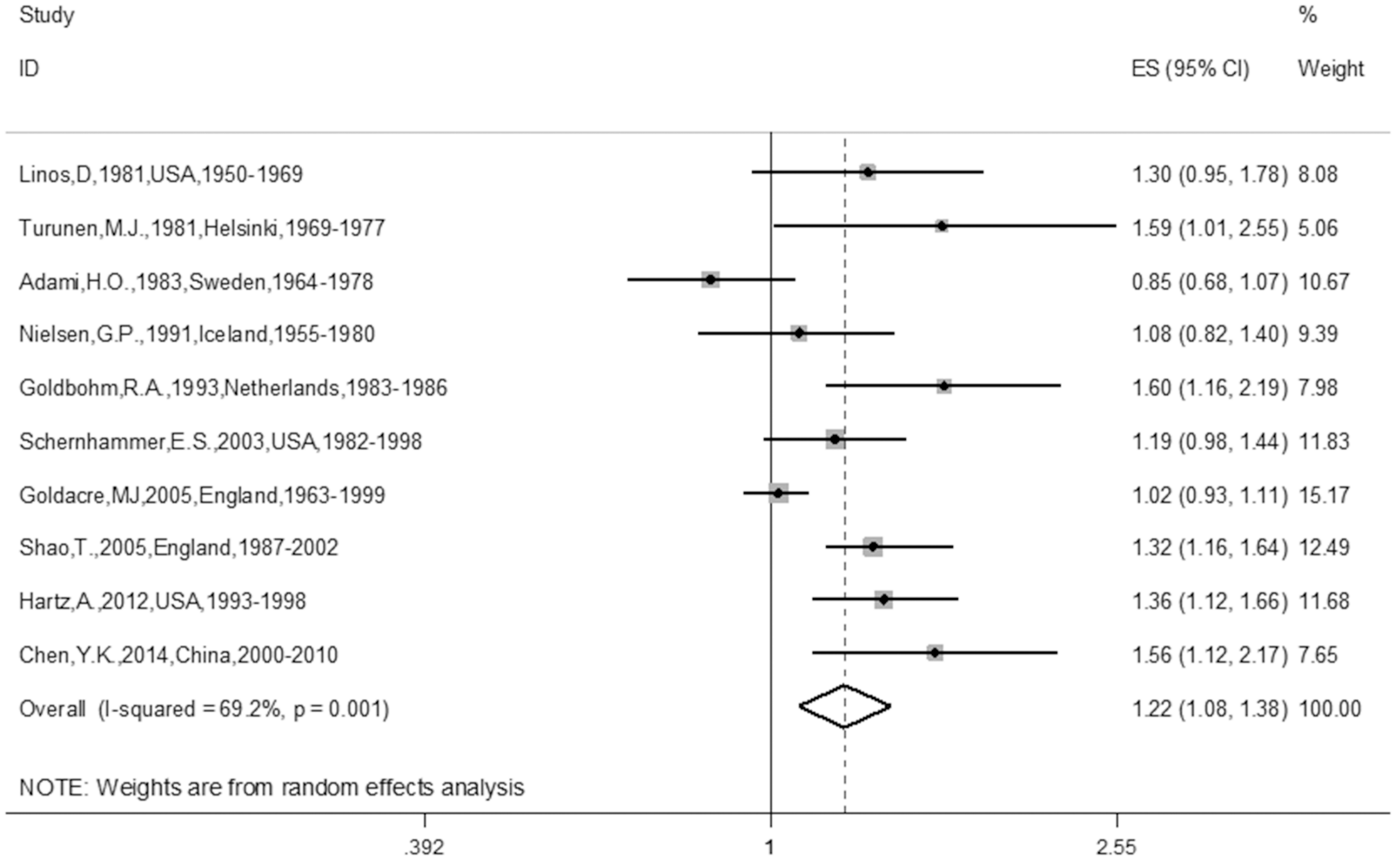
Forest plot of risk of CRC associated with cholecystectomy in general population. Note: CRC represents colorectal cancer.

**Fig 3:**
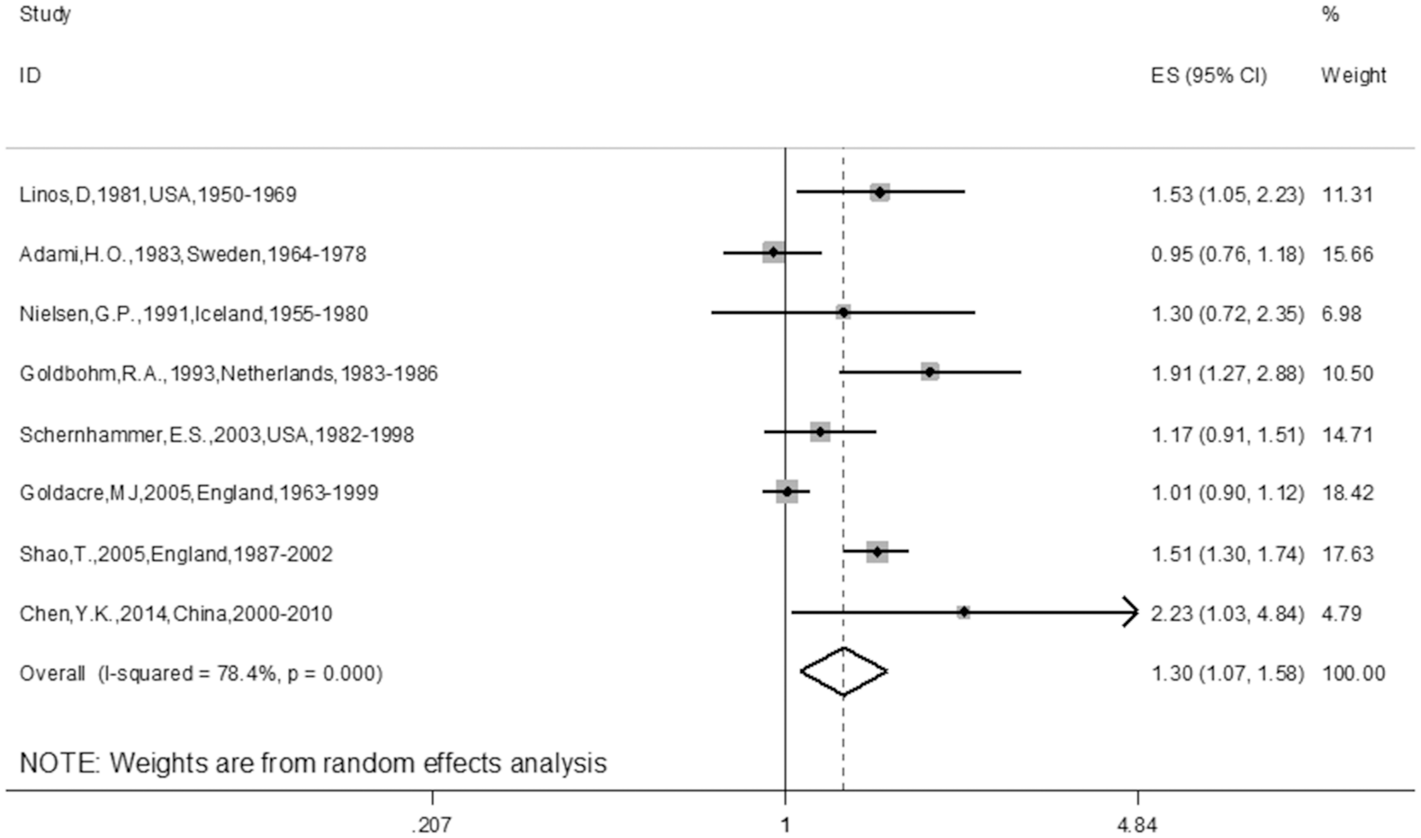
Forest plot of risk of CC associated with cholecystectomy in general population. Note: CC represents colon cancer.

**Fig 4:**
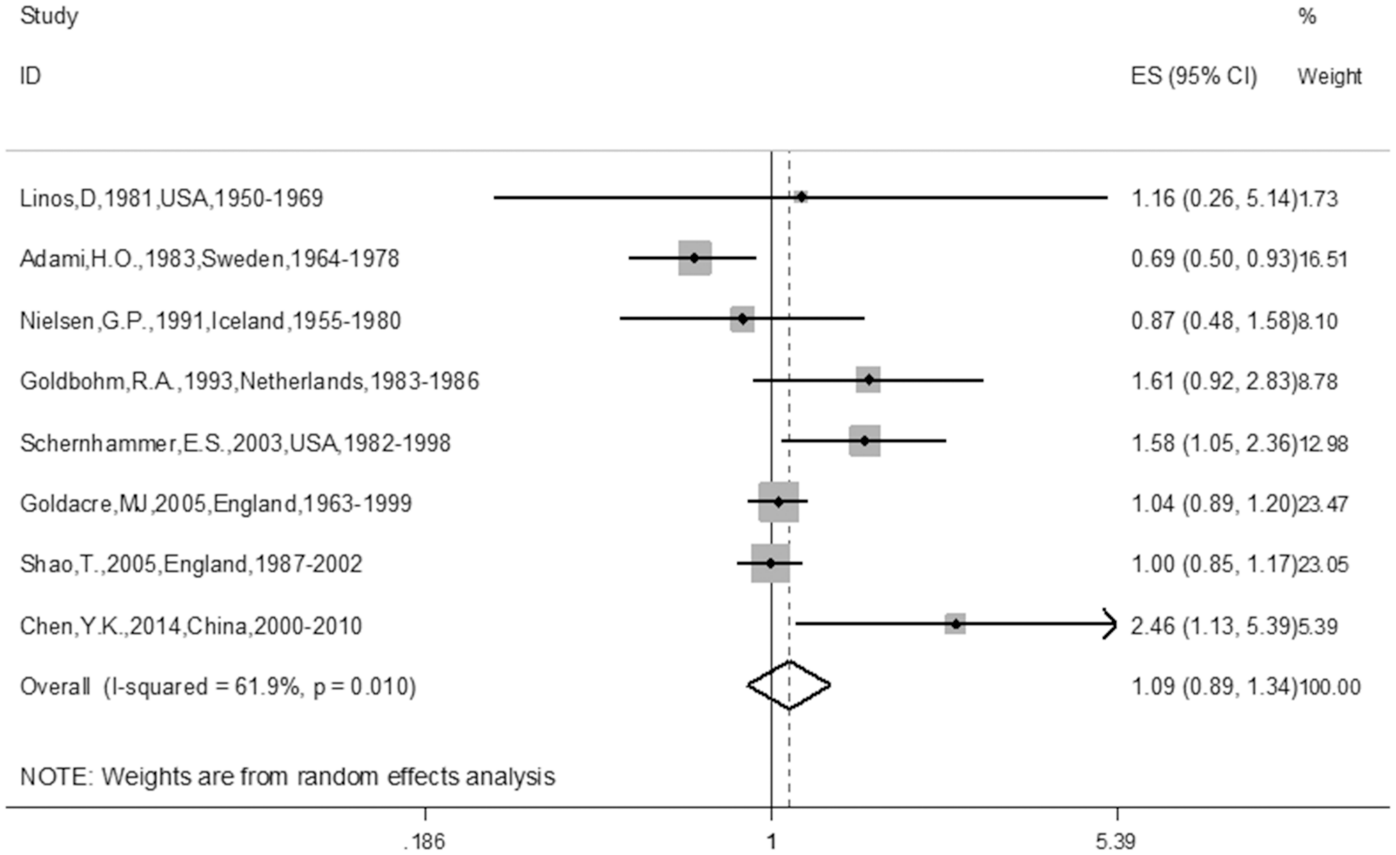
Forest plot of risk of RC associated with cholecystectomy in general population. Note: RC represents rectum cancer.

The meta-analysis of the 10 cohort studies, which included 524,649 targets, demonstrated an increased risk for CRC among individuals who had undergone cholecystectomy (RR 1.22; 95% CI 1.08–1.38). The heterogeneity of the studies was significant (I^2^ = 69.2%, P = 0.001) (Fig 2). In addition, we also found a promising increased risk for CC (RR 1.30, 95% CI 1.07–1.58) (Fig 3) in the sub-group analysis of the CC group. Furthermore, after combining the ascending colon cancer (ACC), transverse colon cancer (TCC), sigmoid colon cancer (SCC) and descending colon cancer (DCC) patients, we found a positive relationship with cholecystectomy (RR 1.18, 95% CI 1.01–1.26). Furthermore, after combining the ACC and DCC patients, a positive relationship with cholecystectomy was also found (RR 1.18, 95% CI 1.01–1.26). There was also a positive relationship between cholecystectomy and ACC (RR 1.18, 95% CI 1.01–1.26) (Table 2) in the sub-group analysis of the tumor location in the colon. There was no relationship between cholecystectomy and RC (RR 1.09; 95% CI 0.89–1.34) (Fig 4). Additionally, there was no relationship between gender and CC or RC; there was only a tendency toward an increase in the female group because the 95% CI included the null number “1” exactly. However, there was a positive relationship between female gender and CRC (RR 1.17; 95% CI 1.03–1.34) (Table 2). Furthermore, we also conducted meta-analyses based on the study region. Of the 10 trials, only one article was from China, the other 9 articles were from Europe or America, namely, Western countries. The sub-group analysis of the study regions revealed that a positive relationship between cholecystectomy and the risk of CRC was found for not only the Western countries group (RR 1.20; 95% CI 1.05–1.36) but also for the America group (RR 1.28; 95% CI 1.12–1.45), but no relationship was observed between cholecystectomy and the risk of CRC in the European countries group (RR 1.16; 95% CI 0.98–1.38) (Table 2). In addition, we found similar results for the main analysis of CRC (RR 1.24; 95% CI 1.09–1.42), the main analysis of CC (RR 1.37; 95% CI 1.12–1.69), and the main analysis of RC (RR 1.15; 95% CI 0.86–1.54) in the sub-group analysis of the NOS ≥7 group (Table 2). Finally, we also did sub-group analyses of follow-up years≥10 and≥ 5 groups, similar results were found between the two sub-group analyses (RR1.17; 95%CI 1.02–1.33 and RR 1.19; 95%CI 1.05–1.35) (Table 2).

**Table 2:** Sub-group analyses of the associations between cholecystectomy and CRC, CC and RC risks.

	CRC				CC				RC			
	Studies	Cases	RR (95% CI)	P (I^2^%)	Studies	Cases	RR (95% CI)	P (I^2^%)	Studies	Cases	RR (95% CI)	P (I^2^%)
Sex												
male	6	1,751	1.26 (0.93–1.70)	0.012 (68.9)	5	810	1.37 (0.93–2.03)	0.010 (69.7)	5	663	1.06 (0.75–1.50)	0.126 (44.3)
female	8	3,327	1.17 (1.03–1.34)	0.054 (51.5)	6	2,215	1.25 (1.06–1.49)	0.093 (47.0)	6	787	1.02 (0.74–1.39)	0.038 (57.5)
overall	14	5,583	1.19 (1.06–1.35)	0.007 (57.4)	11	3,345	1.31 (1.11–1.56)	0.005 (60.6)	11	1,647	1.03 (0.84–1.27)	0.040 (47.3)
female (a)	6	1,705	1.05 (0.91–1.20)	0.045 (55.3)	5	2,141	1.29 (1.03–1.60)	0.058 (56.1)	NA	NA	NA	NA
Location												
AC	NA	NA	NA	NA	8	215	1.18 (1.11–1.26)	0.134 (36.9)	NA	NA	NA	NA
DC	NA	NA	NA	NA	3	158	1.12 (0.81–1.56)	0.230 (32.1)	NA	NA	NA	NA
AC+DC	NA	NA	NA	NA	11	373	1.18 (1.11–1.26)	0.167 (29.3)	NA	NA	NA	NA
AC+TC+DC+SC	NA	NA	NA	NA	13	417	1.18 (1.11–1.26)	0.283 (16.0)	NA	NA	NA	NA
Region												
USA	3	1,664	1.28 (1.12–1.45)	0.631 (0.0)	NA	NA	NA	NA	NA	NA	NA	NA
Europe	6	3,989	1.16 (0.98–1.38)	0.001 (74.5)	NA	NA	NA	NA	NA	NA	NA	NA
Western counties	9	5,653	1.20 (1.05–1.36)	0.001 (68.7)	NA	NA	NA	NA	NA	NA	NA	NA
NOS≥7	8	5,395	1.24 (1.09–1.42)	0.014 (60.2)	7	3,025	1.37 (1.12–1.69)	0.006 (66.9)	7	1,462	1.15 (0.86–1.54)	0.005 (67.3)
Follow-up years												
≥10	8	4,237	1.17(1.02–1.33)	0.005(65.8)	NA	NA	NA	NA	NA	NA	NA	NA
≥5	9	4,715	1.19(1.05–1.35)	0.002(67.4)	NA	NA	NA	NA	NA	NA	NA	NA

Notes: CRC: colorectal cancer; CC: colon cancer; RC: rectum cancer; AC: ascending colon; TC: transverse colon; DC: descending colon; SC: sigmoid colon. Overall: men and women combined; ACC: ascending colon cancer; TCC: transverse colon cancer; DCC: descending colon cancer; SCC: sigmoid colon cancer; RR: relative risk; CI: confidence interval; the P and I^2^ values represent the heterogeneity; Western countries: USA and Europe countries combined; NA: data not applicable. The number of samples may not be equal to the total number because the authors did not describe the specific number of each cancer site in some of the articles. Female (a) indicates the results after excluding the articles with only females. NOS≥7: high quality study.

### Sensitivity analysis

**Fig 5:**
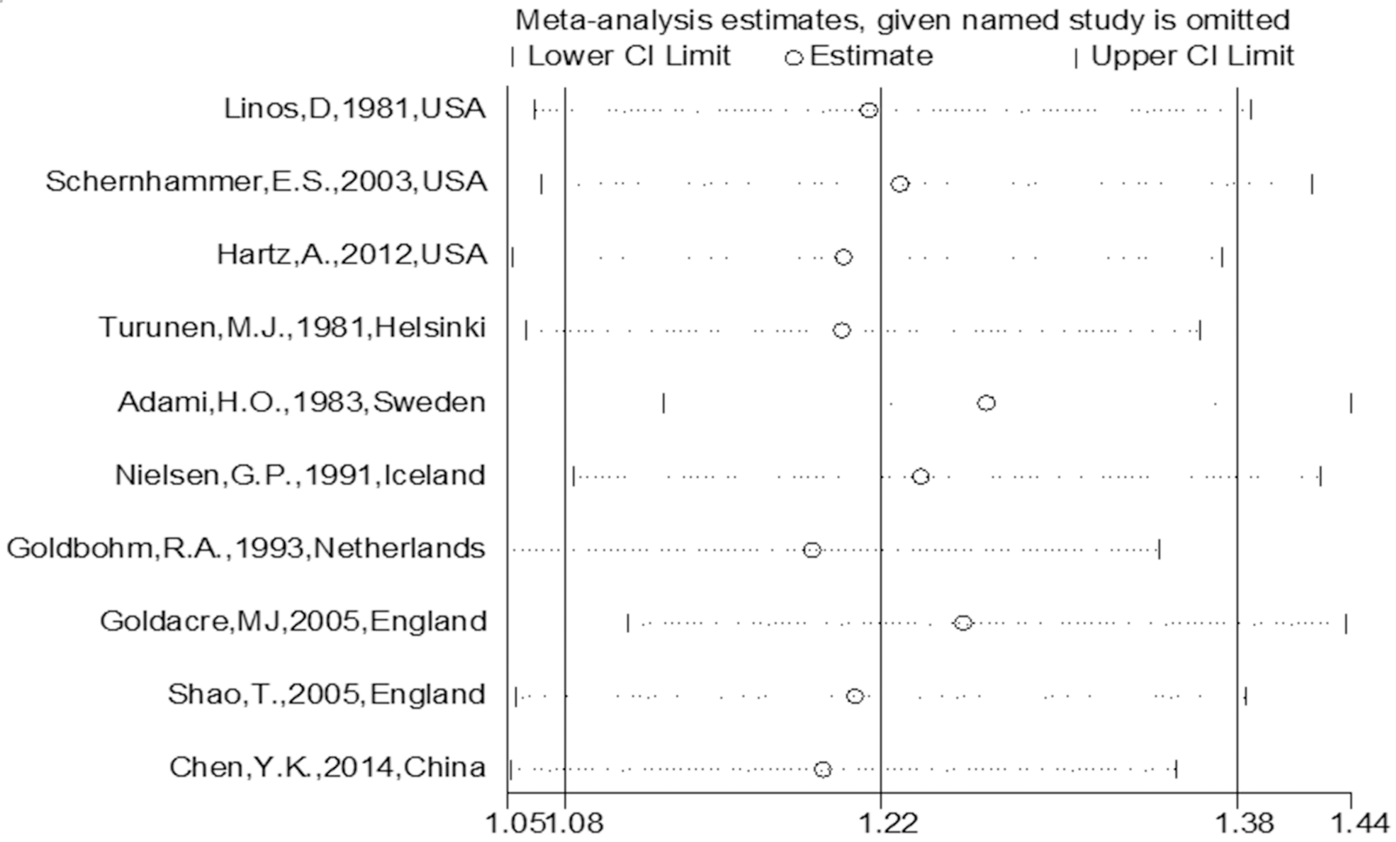
Sensitivity analysis of the association between cholecystectomy and CRC risk in general population. Note: CRC represents colorectal cancer.

To evaluate the robustness of the study, a sensitivity analysis was conducted by excluding one study per iteration to recalculate the pooled results of the primary analysis. The outcome revealed a steady combined result (Fig 5).

### Publication bias

**Fig 6:**
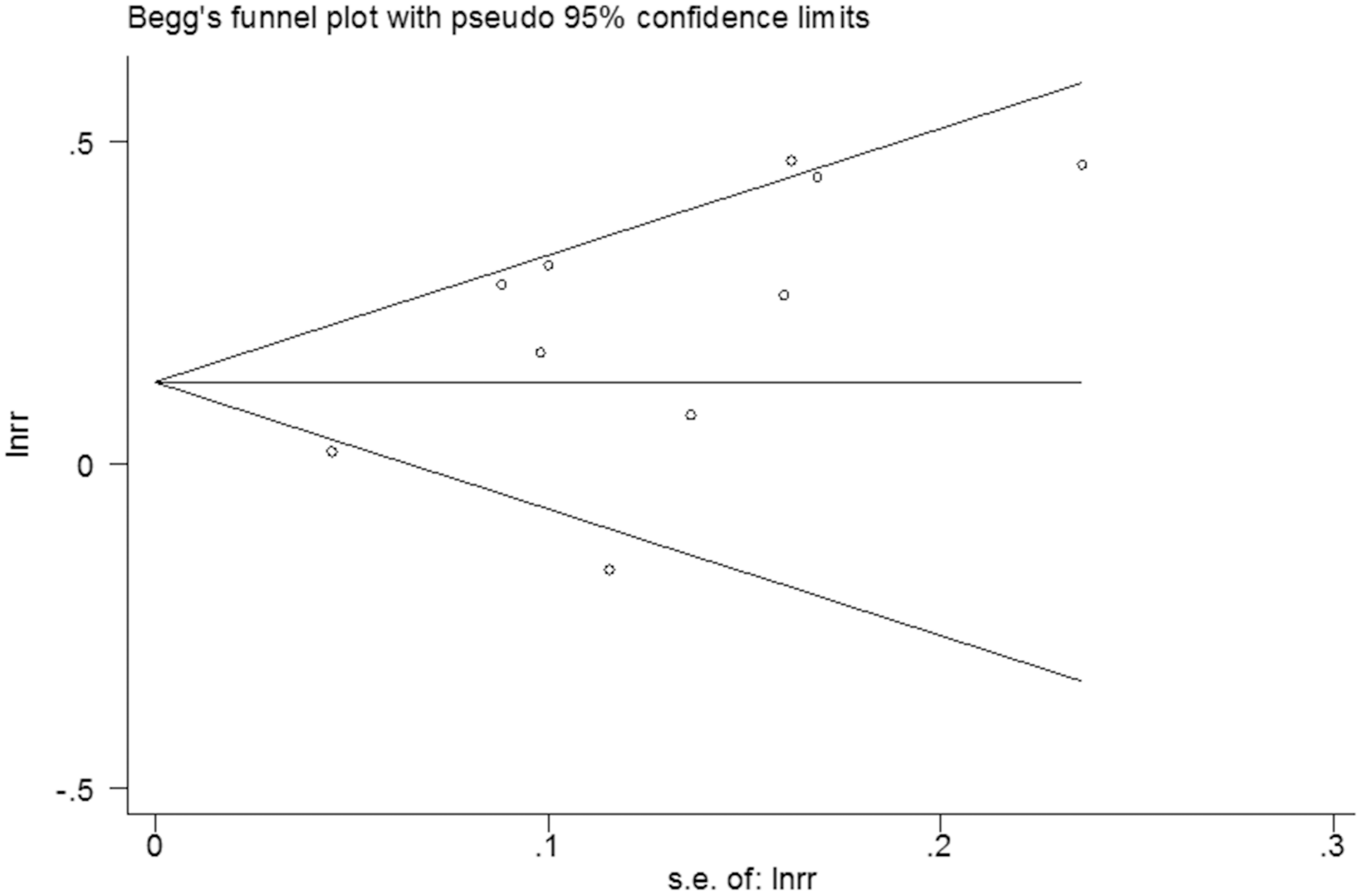
Begg’s funnel plot of the 10 cohort studies.

**Fig 7:**
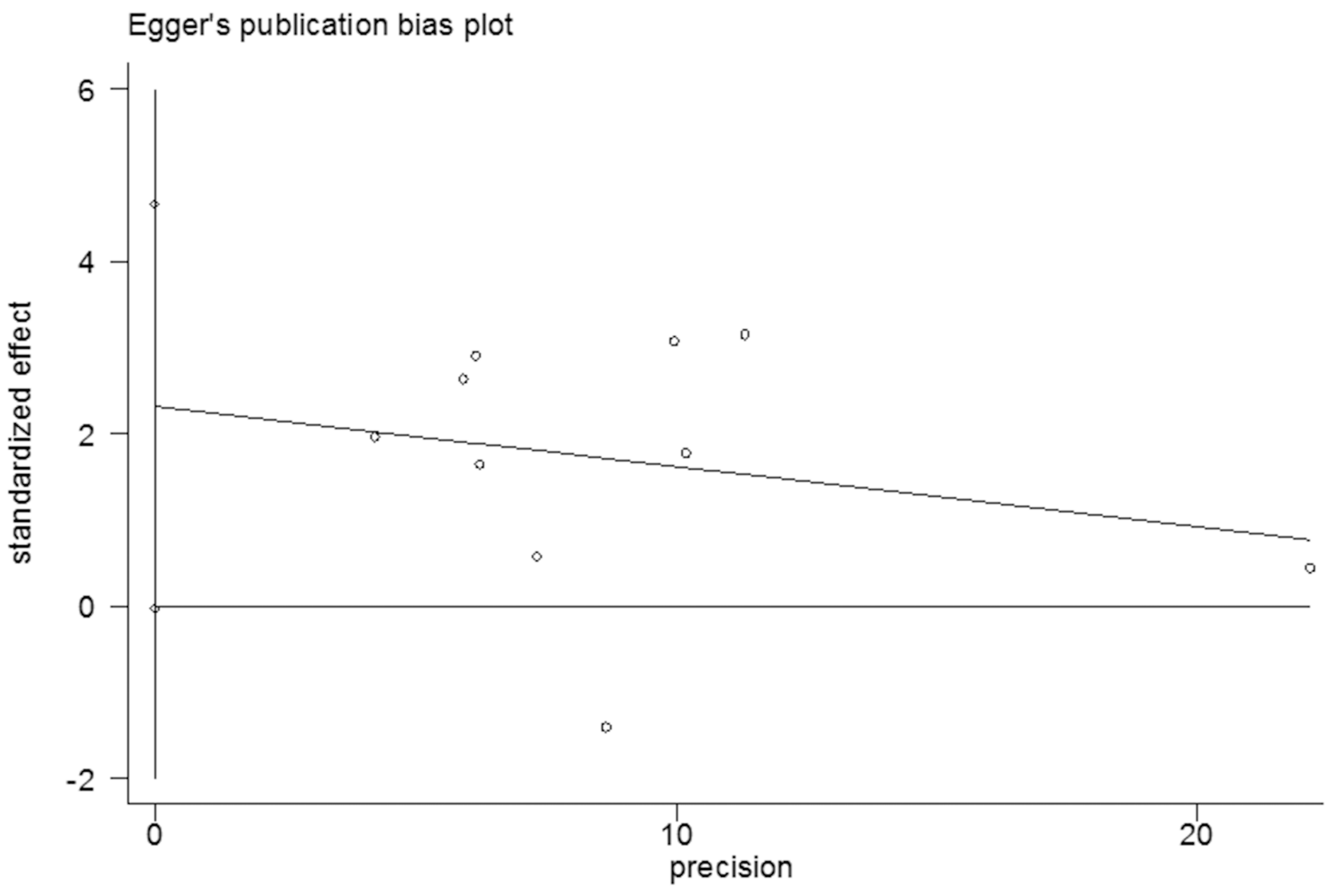
Egger’s publication bias plot of the 13 cohort studies.

No evidence of publication bias among the studies was observed using the Begg rank correlation test and Egger linear regression test [Begg, Pr > |z| = 0.152; Egger, P>|t| = 0.053; 95% CI -0.032–4.66] (Figs 6 and 7).

## Discussion

By searching the PubMed and EMBASE databases, a meta-analysis was performed to describe the effects of cholecystectomy on the risk of CRC using data derived from only cohort studies. The primary results of our study demonstrated that an increased risk for CRC was found among individuals who had undergone cholecystectomy, which is consistent with previous analyses [14–16]. Additionally, we found a promising increased risk for CC, which is in agreement with the North American Association of Central Cancer Registries (NAACCR) reported incidence for CC [40]. In contrast, Chiong’s study [41] found no relationship between cholecystectomy and RC. In addition, when we combined the patients with ACC and DCC with or without the patients with TCC and SCC, we found a positive relationship with cholecystectomy. Furthermore, a positive relationship between cholecystectomy and ACC was discovered in the sub-group analysis of the tumor location in the colon, which is consistent with Reid’s study [15] but contradicts the results of two other papers [17, 31]. In addition, we also found a positive relationship between female gender and CRC only. Furthermore, there was also a positive relationship between cholecystectomy and the risk of CRC in the Western countries group. The sensitivity analysis suggested a stable and robust combined result. No publication bias was observed based on the Begg and Egger tests.

It is well known that gene mutations, polyp disease and the long-term effects of anaerobic bacteria (particularly clostridium) on intestinal mucosa play important roles in the pathogenesis of CRC [42, 43]. Furthermore, the main physiological function of the gallbladder is to store and concentrate bile acids (BAs) [44], which is thought to provide a buffer for the effect of bile on the intestinal tract. Due to the rising incidence of GBD [45], the number of cholecystectomies have also increased. Thus, the physiological properties of BAs may also change postoperatively to some extent. Several experimental studies [46, 47] have shown that BAs drain into the digestive system continuously due to a loss of bile storage and the relaxation of the Oddi sphincter following cholecystectomy. In addition, the composition and secretion of BAs also changes [47]. These changes may promote the metabolites of BAs, namely, secondary BAs, to repeatedly stimulate the intestinal mucosa [48, 49]. Consequently, BAs and the products of the intestinal micro flora also correspondingly elevate the risk for CRC in post-cholecystectomy patients [50].

Secondary BAs, particularly lithocholic acid (LCA) and deoxycholic acid (DCA), are regarded as potential tumor-promoting agents in the etiology of CRC [51] due to their similar molecular structure with carcinogenic polycyclic aromatic hydrocarbons. In fact, BAs were first thought to be carcinogenic in 1940 [52]. Furthermore, LCA [53] and DCA [54] are also considered toxic endobiotics. Additionally, Ajouz et al. [55] and Bernstein [56] showed that secondary BAs contribute to the development of CRC through multiple biochemical and physiological aspects. In addition, previous studies [57, 58] proved that the content of BA and cholesterol in feces was significantly higher in experimental or clinical CRC cases than in normal individuals, and these findings were more common in individuals from Western countries [59]. Furthermore, high physiological levels of BAs can lead to apoptosis resistance, genomic instability and ultimately, to cancer [60]. BA hydrophobicity is implicated in the promotion of CRC [61]. Furthermore, studies have revealed that colon polyps arising from the glandular epithelium, another major cause of CRC [62], are also positively affected by secondary BAs [63, 64]. Hence, these interrelated pathways represent the possible effects of cholecystectomy on CRC.

Additionally, diet and lifestyle also affect the carcinogenesis of CRC [65]. More than 40 years ago, Berg et al. [66] concluded that consuming a Western diet leads to a higher risk of CRC, even in low-risk populations. A recently published paper [9] highlighted a similar conclusion. The hazard of consuming a diet high in fat, animal fat and animal protein lies in the excess stimulation biliation and bile excretion [67]. In individuals with CRC, in addition to an increase in the amount of fecal anaerobic bacteria, there is also an increase in the dehydrogenase activity of bacterial β-glucuronidase, 7α-decarboxylase and cholesterol [68]. β-glucuronidase converts non-toxic substances into harmful substances. Furthermore, 7α-decarboxylase converts BA into deoxycholic acid, which is a carcinogen. Moreover, cholesterol is primarily metabolized into BAs in liver cells [69]. Insulin can induce the synthesis of HGM-CoA reductase in the liver, increasing the synthesis of cholesterol. Therefore, obesity linked to insulin resistance can lead to hyperinsulinemia [70], which arises from high physiological levels of BAs. Furthermore, the steroid hormone estradiol is a precursor to cholesterol and affects the synthesis of bile in liver cells [71]. These findings may explain the higher risk of CRC following cholecystectomy in the Western or Western diet-consuming population as well as the increased risk in females *versus* males. Moreover, no relationship was observed between cholecystectomy and RC in this study.

There are several strengths to this study. Cohort studies are able to fully prove causality, which is not possible in observational studies [72]. Therefore, we excluded case-control and epidemiological observational studies and only included cohort studies. Additionally, the included studies had followed up the participants for a long period of time. Most studies provided more RRs or SIRs, which were used for the sub-group analyses. Confounders were estimated in most of the studies. Eight studies were considered high quality. All of the included studies included a large sample size, increasing the accuracy of the outcomes. Furthermore, because almost all of the accepted studies were performed in Western countries and only one study was conducted in Asia [39], we also performed a sub-group analysis for the Western countries group. The results were favorable (RR 1.20; 95% CI 1.05–1.36), indicating that there was a positive relation between cholecystectomy and CRC risk in Western countries. Furthermore, in the sub-group analysis of gender, males in particular introduced heterogeneity. These factors will be assessed in future studies.

There are also some limitations to our meta-analysis. First, in sub-group analyses, we found a positive relationship between female gender and cholecystectomy for CRC and CC but not for RR (Table 2). After removed the articles [29, 38] including only women, there was still a positive relationship between CC and cholecystectomy in females; however, the effect of female gender on the risk of CRC after cholecystectomy was not observed. Several studies [73–75] have shown that estrogen can reduce the risk of CRC, whereas other studies [76–78] have also shown that testosterone can increase the risk of CRC. However, none of these findings can verify our results. Hence, we speculate that variations in the sample size created significant discrepancy in the results. However, there was a significant positive correlation between the incidence of CRC or CC and cholecystectomy in females. Additionally, to determine the source of heterogeneity, we performed a sub-group analysis for the NOS ≥7 group after removing the low quality studies [32, 37]. We observed similar results as the main analysis for the risk of CRC, CC and RC, but the heterogeneity was not eliminated (Table 2). Therefore, the low quality studies did not influence the final results or the heterogeneity. Second, we only included studies on the association between cholecystectomy and CRC risk that were published in English. Studies published in languages other than English were excluded due to language barriers. Furthermore, some studies may have been missed because they were published in books that are not captured in the Internet databases. Therefore, the number of accepted studies was low. Third, some of the studies included unclear or incomplete data, making the data analysis difficult. For this reason, we used the Stata software for the data analysis because we were unable to obtain the original data from the authors. Moreover, some studies only described the relationship between cholecystectomy and CC or RC, without stating the relationship with CRC. Therefore, these data were included in the sub-group analyses for CC or RC. Furthermore, we used a random-effects model to calculate the RR and 95% CI of CRC by combining the data for CC and RC. However, there was significant heterogeneity, particularly in the three main analyses. After combining the ACC, TCC, DCC and SCC patients, the heterogeneity was eliminated in the sub-group analyses for the tumor location in the colon (I^2^ = 16.0%, P = 0.283). Although only one article [39] provided the RRs and 95% CIs for TCC and SCC, heterogeneity was not observed after this study was removed (I^2^ = 29.3%, P = 0.167) (Table 2). Fourth, the subgroup analysis of follow-up year was carried out, and the values of P and I-square were high(Table 2). The sub-group analyses of follow-up years could not explain the source of heterogeneity either. The values of P and I-square of each sub-group were similar and had little difference with the main analysis. Furthermore, we were unable to analyze the influence of age because there was no significant difference in age among the included patients. Fifth, although eight of the ten studies were considered high quality, two were also low quality papers that were included in the study to increase the sample size. However, although the final results were stable, there was significant heterogeneity which we were unable to eliminate. The existence of high heterogeneity and failure to find the root of heterogeneity are important flaws in our analysis. Sixth, we only conducted sub-group analyses for CC, the tumor location in the colon, RC, the study geographical location and sex, without adjusting for other confounders such as age, race, smoking, alcohol, diet, medications, and physical exercise. Considering that lifestyle factors cannot be controlled for among the included patients, these confounding factors were assumed to be equal across the studies. Seventh, although both the morbidity of CRC and the rate of cholecystectomy have increased, there are few relevant cohort studies. Furthermore, only two articles [29, 39] have been published in the last decade, which may be the reasons for the observed heterogeneity and outcomes in the study. Finally, our meta-analysis is not comprehensive because the number of included cohort studies was low, and the quality of the studies was inconsistent. Thus, further studies are needed to perform more comprehensive and higher quality analyses.

## Conclusions

In summary, our meta-analysis proved that cholecystectomy is associated with an increased risk for CRC, CC and ACC, particularly in Western countries. There is no relationship between cholecystectomy and RC. The relationship between gender and CRC, CC or RC is unclear. Further studies on CRC that adhere to strict criteria are needed to further determine the impact of cholecystectomy on the risk of large intestine carcinoma.

## Supporting information

S1 Table PRISMA checklist. (DOC)
