# Correction: Cholecystectomy can increase the risk of colorectal cancer: A meta-analysis of 10 cohort studies

**DOI:** 10.1371/journal.pone.0191587

**Published:** 2018-01-17

**Authors:** Yong Zhang, Hao Liu, Li Li, Min Ai, Zheng Gong, Yong He, Yunlong Dong, Shuanglan Xu, Jun Wang, Bo Jin, Jianping Liu, Zhaowei Teng

There is an error in the second sentence of the Results section of the Abstract. The correct sentence is: According to the Newcastle-Ottawa Scale (NOS), eight papers were considered high quality.

There is an error in the third sentence of the Results section of the Abstract. The correct sentence is: After the data of these 10 studies were combined, an increased risk of CRC was found among the individuals who had undergone cholecystectomy (risk ratio (RR) 1.22; 95% confidence interval (CI) 1.08–1.38).

There is an error in the last sentence under the heading “Literature search and study characteristics” in the Results section. The correct sentence is: The NOS scores for 8 of the included articles were ≥ 7.0, indicating high quality.

There is an error in the fourth sentence of the Discussion section. The correct sentence is: But, contrary to Chiong’s study [41], no relationship between cholecystectomy and RC was found in our study.
