# Supplementary material for: Expression of Concern: Cholecystectomy can increase the risk of colorectal cancer: A meta-analysis of 10 cohort studies
Source: PLoS One. 2024 Mar 8;19(3):e0300484. doi: 10.1371/journal.pone.0300484 (PMC10923401; doi:10.1371/journal.pone.0300484)
Supplement: S1 File — (ZIP) [file pone.0300484.s001.zip › Raw data specification.docx]

1. **Search strategy**: The retrieval method and inclusion criteria were described in the article. The process of literature retrieval can be seen in colorectal cancer retrieval strategy.csv of the attachment.

2. **Search Results**: The retrieved article directory according to the search strategy can be seen in pubmed_results.txt.

3. **Literature inclusion**: Endnote software is used to sort, group, and delete the literature retrieval, as shown in "Cholecystectomy and Colon Cancer.data" and "Cholecystectomy and Colon Cancer.enl". Then, the initial literature for inclusion were identified.

4. **Data extracted**: The raw data extracted can be seen as "original data.xlsx". Originally, 13 articles were included. After submitting for the first time, I will take the reviewer's opinions, and finally enroll in 10 cohort studies by reading the original text in detail, comparing the sample size of the article, and clarifying whether there are duplicates. Detailed data can be found in "sheet6" in "Original Data.xlsx".

5. **Data Analysis**: The included literature data were classified into subgroup analysis according to study region, study gender, colorectal cancer incidence site, follow-up years, etc., and then analyzed by Stata SE 15. Please refer to " sheet7" in "Original Data.xlsx" for detailed data.
